# Tuberculosis Diagnosis, Treatment, and Prevention Services for Children Living with HIV in Low- and Middle-Income Countries: A Multiregional Site Survey

**DOI:** 10.1093/jpids/piaf050

**Published:** 2025-05-28

**Authors:** Katherine Laycock, Karl-Günter Technau, Patricia Lelo, Watsamon Jantarabenjakul, Caroline Yonaba, Jorge Pinto, Michael Menser, Fernanda Maruri, Francesca Odhiambo, Ethel Rambiki, Pélagie Babakazo, Van Lam Nguyen, Madeleine Folquet, Daisy Maria Machado, Nelson Kalema, Guy Muula, Ellen Brazier, Dinh Qui Nguyen, Joycelyn Dame, Marco Tulio Luque, Aggrey Semeere, Brian Eley, Marcel Yotebieng, Azar Kariminia, Vanessa Rouzier, Helen Byakwaga, Olivier Marcy, Leslie A Enane

**Affiliations:** The Ryan White Center for Pediatric Infectious Disease and Global Health, Department of Pediatrics, Indiana University School of Medicine, IN, United States; Empilweni Services and Research Unit (ESRU), Rahima Moosa Mother and Child Hospital, Department of Paediatrics and Child Health, Faculty of Health Sciences, University of The Witwatersrand, Johannesburg, South Africa; Kalembelembe Pediatric Hospital, Unit of Infectious Diseases, Kinshasa, Democratic Republic of Congo; Center of Excellence for Pediatric Infectious Diseases and Vaccines, Department of Pediatrics, Faculty of Medicine, Chulalongkorn University, Bangkok, Thailand; Pediatric Department, Centre Hospitalier Universitaire Yalgado Ouédraogo, Ouagadougou, Burkina Faso; Federal University of Minas Gerais, Belo Horizonte, Brazil; Indiana University Department of Biostatistics and Health Data Science, Indianapolis, United States; Division of Infectious Diseases, Vanderbilt University School of Medicine, Nashville, United States; Family AIDS Care and Education Services, Kenya Medical Research Institute, Kisumu, Kenya; Lighthouse Clinic Trust, Lilongwe, Malawi; Kinshasa School of Public Health, Democratic Republic of Congo; Center for Tropical Diseases, Vietnam National Children’s Hospital, Hanoi, Vietnam; CHU Cocody, Service Pédiatrie, Abidjan, Côte d’Ivoire; Escola Paulista de Medicina - Federal University of São Paulo (EPM-UNIFESP), São Paulo, Brazil; Infectious Diseases Institute, College of Health Sciences, Makerere University, Kampala, Uganda; Center for Infectious Disease Research in Zambia, Lusaka, Zambia; City University of New York, Institute for Implementation Science in Population Health, New York, United States; Infectious Diseases Department, Children’s Hospital 2, Ho Chi Minh City, Vietnam; University of Ghana Medical School and Korle Bu Teaching Hospital, AccraGhana; Instituto Hondureño de Seguridad Social, Tegucigalpa, Honduras; Infectious Diseases Institute, College of Health Sciences, Makerere University, Kampala, Uganda; Red Cross War Memorial Children’s Hospital and the Department of Paediatrics and Child Health, University of Cape Town, Cape Town, South Africa; Division of General Internal Medicine, Department of Medicine, Albert Einstein College of Medicine, The Bronx, New York, United States; The Kirby Institute, UNSW Sydney, Australia; Les Centres GHESKIO, Port-au-Prince, Haiti; Mbarara University of Science and Technology, Mbarara, Uganda; University of Bordeaux, Inserm U1219 Bordeaux Population Health, IRD EMR271 GHiGS, Bordeaux, France; The Ryan White Center for Pediatric Infectious Disease and Global Health, Department of Pediatrics, Indiana University School of Medicine, IN, United States; Indiana University Center for Global Health Equity, Indianapolis, United States

**Keywords:** pediatrics, TB, HIV, diagnostic access, TB preventive therapy

## Abstract

**Background:**

Tuberculosis (TB) remains a leading cause of morbidity and mortality for children living with HIV (CLHIV), with gaps in TB screening, diagnostics, management, and TB preventive therapy (TPT). We investigated reported practices in these domains at sites caring for CLHIV in low- and middle-income countries (LMICs) within the International Epidemiology Databases to Evaluate AIDS (IeDEA) consortium.

**Methods:**

We implemented a site survey from September 2020 to February 2021, querying pre-pandemic practices. This analysis included sites in LMICs providing care for CLHIV that diagnosed TB in 2019. We analyzed responses using descriptive statistics and assessed regional differences using Fisher’s exact or chi-square tests.

**Results:**

Of 238 IeDEA sites, 227 (95%) responded and 135 met the inclusion criteria. Most (90%) reported screening for TB at HIV care enrollment. Access to diagnostics varied significantly by region, including nucleic acid amplification testing (NAAT, range 67-100%), mycobacterial culture (range 43%-83%), and drug susceptibility testing (range 30%-82%) (*P* < .001). On-site TB treatment was high (90%). Reported stock-outs occurred for isoniazid (23/116, 20%) and other TB medications (11/114, 9.6%, range 0%-33%, *P* = .008). TPT provision ranged 50%-100% (*P* < .001). Six months of isoniazid was the most common TPT regimen for children (88%). Shorter TPT regimens were uncommon (0.9%-2.8%), as were regimens for multidrug-resistant TB exposure (4.6%).

**Conclusions:**

Overall reported availability of NAAT and integrated TB/HIV treatment for CLHIV cared for at these IeDEA sites in LMICs is encouraging but varies by context. Heterogeneous implementation gaps remain—particularly for drug susceptibility testing, TPT delivery, and TPT regimens—which may impede TB prevention, management, and successful outcomes for CLHIV, warranting continued close attention over time and as global TB care guidelines and services evolve.

## Teaser

In this global survey of tuberculosis (TB) management practices at 135 sites providing care for children living with HIV, we identified encouraging overall availability of some key tools. However, heterogeneous implementation gaps remain, with implications for TB care and prevention.

## Introduction

Among children living with HIV (CLHIV), tuberculosis (TB) is a major cause of morbidity and mortality.^1-3^ In 2022, an estimated 31 000 CLHIV (<15 years of age) died from TB worldwide.^1^ CLHIV experience a higher risk of TB mortality compared with those without HIV, including after initiation of antiretroviral therapy (ART).^2,4^

Childhood TB is severely underdiagnosed when compared to TB in older age groups. Reasons for this include limited recognition of TB manifestations in children; the inability of young children to produce sputum for evaluation; and the pauci-bacillary nature of TB in children, which decreases the sensitivity of TB diagnostics. It is estimated that over 95% of TB mortality in children is due to a lack of TB diagnosis.^5^ Access to essential diagnostic tests and modalities, including molecular diagnostics such as Xpert MTB/RIF and Xpert Ultra, gastric aspiration, induced sputum, and others, is critically needed to improve pediatric case-finding. Screening and diagnostic difficulties are further compounded in CLHIV, particularly in those under age 5 and in those with advanced HIV, as clinical and radiographic features of TB have limited sensitivity in these vulnerable children.^4^ Integrated TB and HIV care, with co-located diagnosis and treatment, provides a framework to support optimal outcomes for CLHIV, but implementation has also been limited. A previous multisite survey by the International Epidemiology Databases to Evaluate AIDS (IeDEA) found gaps in the availability of critical diagnostics, and a need to develop capacity at ART programs to diagnose or exclude TB in CLHIV.^6^

Iterative data are needed regarding the management and prevention of TB in CLHIV across global settings. The World Health Organization (WHO) provides valuable guidelines on the screening, diagnosis, management, and prevention of HIV-associated TB; however, gaps between policy and practice implementation have long hampered efforts to end TB, especially among children.^7,8^ We sought to ascertain data from routine operations across the global IeDEA consortium of HIV care programs. Reported practices in pediatric TB care at these sites provide critical data regarding implementation gaps that deserve ongoing attention and action to achieve optimal outcomes for CLHIV.

## Methods

We conducted a site survey of HIV clinics with a focus on pediatric TB as part of a general IeDEA-wide site assessment. Established in 2006, IeDEA is an NIH-funded global consortium of HIV care sites across 44 countries in seven geographic regions: Asia Pacific; the Caribbean, Central, and South America; North America; Central Africa; East Africa, Southern Africa; and West Africa. IeDEA serves to collect, harmonize, and analyze observational data from global sites serving more than 2 million individuals living with or at risk for HIV, to address key research questions in the evolving HIV/AIDS epidemic. In 2019, approximately 40 000 CLHIV (<15 years of age) were actively engaged in HIV care at IeDEA sites. The IeDEA consortium periodically collects data from participating HIV care sites regarding service availability across sites and over time.^9^ This has been accomplished through site surveys delivered to partner sites across the consortium.

As part of IeDEA’s 2020 site assessment, questions were included detailing service availability related to pediatric TB diagnosis and treatment. The process for developing and implementing the site assessment has been previously described.^9^ In this process, IeDEA’s technical working groups reviewed the content of the previous general site assessment survey and made recommendations for items that should be retained, retired, modified, or added to address evidence gaps. The updated survey was iteratively refined to standardize the format of new question items and implement best practices in survey design as well as lessons learned from previous site assessments.^9^

The site assessment survey was self-administered in English or French, using paper or online Research Electronic Data Capture (REDCap) data entry. REDCap, a web-based software platform for secure data capture in research studies, provides a user-friendly interface for data capture; audit capabilities; and automated export routines.^10,11^ Site partners selected staff with in-depth knowledge about care and services provided at the site to respond to queries in each site assessment domain.^9^ The survey was implemented from September 2020 to February 2021. Given multiple disruptions and alterations to services during the COVID pandemic, sites were asked to refer to pre-COVID practices.

For this analysis of survey items regarding services related to pediatric TB diagnosis, prevention, and treatment, inclusion was limited to sites in low- and middle-income countries (LMICs) that provided care for CLHIV and reported diagnosing TB in 2019. Survey responses were analyzed using descriptive statistics. Differences by IeDEA region were assessed using Fisher’s exact tests (for variables with at least one expected cell frequency < 5) or chi-square tests (for variables with expected cell frequencies ≥ 5).

## RESULTS

### Characteristics of Participating Pediatric ART Programs

**Figure 1.**
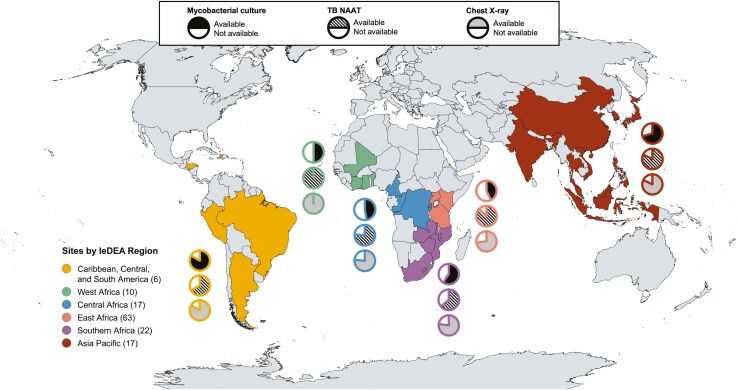
Country Locations of IeDEA Sites Included in this Analysis, by IeDEA Region. Reported Availability of Key Diagnostics (Mycobacterial culture, TB NAAT, and chest X-ray) are Presented by IeDEA Region. Map created using mapchart.net. Abbreviations: IeDEA, International epidemiology Databases to Evaluate AIDS; NAAT, nucleic acid amplification testing; TB, tuberculosis.

We surveyed 238 IeDEA sites providing care to people living with HIV (PLHIV), and 227 sites (95.4%) responded. Of these, 177 sites (74.3%) reported diagnosing TB in 2019. In this study, we analyzed data from the 135 ART programs (57.1%) in LMICs that both provided care for CLHIV and reported diagnosing TB in 2019 (Figure 1). Most sites (86.7%) also provided care to adults with HIV. Characteristics of the 135 sites, located in 32 countries across six IeDEA regions, are shown in Table 1. Sixty-eight sites (50.4%) were health centers, 15 (11.1%) were district hospitals, and 45 (33.3%) were regional, provincial, or university hospitals. Sites were in urban (29.6%), periurban (44.4%), and rural settings (25.9%).

**Table 1.** Site Characteristics of 135 Clinics Serving Children Living With HIV in the Global IeDEA Consortium Which Diagnosed TB in 2019—Overall and by IeDEA Regions.

Characteristic *n* (%)	All	Asia-Pacific	Caribbean, Central, and South America	Central Africa	East Africa	Southern Africa	West Africa
Clinics	135	17 (12.6%)	6 (4.4%)	17 (12.6%)	63 (46.7%)	22 (16.3%)	10 (7.4%)
Number of countries, *n*	32	9	5	5	3	6	4
Setting Urban Periurban Rural	40 (29.6%) 60 (44.4%) 35 (25.9%)	7 (41.2%) 10 (58.8%) 0 (0%)	6 (100%) 0 (0%) 0 (0%)	7 (41.2%) 8 (47.1%) 2 (11.8%)	0 (0%) 33 (52.4%) 30 (47.6%)	15 (68.2%) 4 (18.2%) 3 (13.6%)	5 (50.0%) 5 (50.0%) 0 (0%)
Level of care Health center District hospital Regional/provincial/university hospital Unknown	68 (50.4%) 15 (11.1%) 45 (33.3%) 7 (5.2%)	2 (11.8%) 0 (0%) 15 (88.2%) 0 (0%)	0 (0%) 0 (0%) 6 (100%) 0 (0%)	9 (52.9%) 0 (0%) 8 (47.1%) 0 (0%)	44 (69.8%) 12 (19.0%) 6 (9.5%) 1 (1.6%)	11 (50.0%) 1 (4.5%) 4 (18.2%) 6 (27.3%)	2 (20.0%) 2 (20.0%) 6 (60.0%) 0 (0%)
Patients treated Children (0-9 years) Adolescents (10-24 years) Adults (≥20 years)	135 (100%) 135 (100%) 117 (86.7%)	17 (100%) 17 (100%) 10 (58.8%)	6 (100%) 6 (100%) 5 (83.3%)	17 (100%) 17 (100%) 17 (100%)	63 (100%) 63 (100%) 62 (98.4%)	22 (100%) 22 (100%) 19 (86.4%)	10 (100%) 10 (100%) 4 (40.0%)
Availability of routine TB screening^1^ TB infection Screening at enrollment Screening at follow-up In HIV clinic In same health facility Offsite only Not available For children < 10 years TB disease Screening at enrollment Screening at follow-up In HIV clinic In same health facility Offsite only Not available For children < 10 years	52 (38.5%) 39 (28.9%) 26 (19.3%) 20 (14.8%) 50 (37.0%) 52 (38.5%) 121 (89.6%) 106 (78.5%) 20 (14.8%) 8 (5.9%) 1 (0.7%) 119 (88.2%)	5 (29.4%) 8 (47.1%) 3 (17.7%) 1 (5.9%) 5 (29.4%) 12 (70.6%) 14 (82.4%) 15 (88.2%) 2 (11.8%) 0 (0%) 0 (0%) 15 (88.2%)	4 (66.7%) 1 (16.7%) 2 (33.3%) 2 (33.3%) 1 (16.7%) 3 (50.0%) 5 (83.3%) 4 (66.7%) 0 (0%) 2 (33.3%) 0 (0%) 4 (66.7%)	6 (35.3%) 5 (29.4%) 0 (0%) 4 (23.5%) 8 (47.1%) 2 (11.8%) 14 (82.4%) 13 (76.5%) 1 (5.9%) 2 (11.8%) 1 (5.9%) 10 (58.8%)	24 (38.1%) 18 (28.6%) 13 (20.6%) 9 (14.3%) 23 (36.5%) 24 (38.1%) 59 (93.7%) 52 (82.5%) 10 (15.9%) 1 (1.6%) 0 (0%) 61 (96.8%)	8 (36.4%) 5 (22.7%) 6 (27.3%) 1 (4.6%) 10 (45.5%) 6 (27.3%) 22 (100%) 17 (77.3%) 5 (22.7%) 0 (0%) 0 (0%) 20 (90.9%)	5 (50.0%) 2 (20.0%) 2 (20.0%) 3 (30.0%) 3 (30.0%) 5 (50.0%) 7 (70.0%) 5 (50.0%) 2 (20.0%) 3 (30.0%) 0 (0%) 9 (90.0%)
On-site TB disease treatment Pediatric patients Adult patients No on-site treatment	122 (90.4%) 114 (84.4%) 9 (6.7%)	16 (94.1%) 9 (52.9%) 1 (5.9%)	5 (83.3%) 5 (83.3%) 1 (16.7%)	14 (82.4%) 14 (82.4%) 2 (11.8%)	61 (96.8%) 62 (98.4%) 1 (1.6%)	18 (81.8%) 19 (86.4%) 2 (9.1%)	8 (80.0%) 5 (50.0%) 2 (20.0%)
Clinics with co-located pharmacy Pharmacies with TB medications available to dispense^2^ Isoniazid Rifapentine Other TB medications Pharmacies experiencing TB medication stockouts of > 1 week^3^ Isoniazid Rifapentine Other TB medications	131 (97.0%) 116 (88.6%) 28 (21.4%) 114 (87.0%) 23 (19.8%) 3 (10.7%) 11 (9.6%)	16 (94.1%) 15 (93.8%) 4 (25.0%) 15 (93.8%) 3 (20.0%) 0 (0%) 1 (6.7%)	5 (83.3%) 3 (60.0%) 1 (20.0%) 3 (60.0%) 1 (33.3%) 0 (0%) 1 (33.3%)	17 (100%) 13 (76.5%) 5 (29.4%) 15 (88.2%) 3 (23.1%) 2 (40.0%) 5 (33.3%)	63 (100%) 60 (95.2%) 11 (17.5%) 59 (93.7%) 14 (23.3%) 0 (0%) 3 (5.1%)	20 (90.9%) 20 (100%) 6 (30.0%) 18 (90.0%) 2 (10.0%) 1 (16.7%) 1 (5.6%)	10 (100%) 5 (50.0%) 1 (10.0%) 4 (40.0%) 0 (0%) 0 (0%) 0 (0%)
Fees charged to patients for TB medications (other than insurance copays) Yes No Not applicable	4 (3.0%) 127 (94.1%) 4 (3.0%)	0 (0%) 17 (100%) 0 (0%)	0 (0%) 6 (100%) 0 (0%)	2 (11.8%) 15 (88.2%) 0 (0%)	2 (3.2%) 59 (93.7%) 2 (3.2%)	0 (0%) 21 (95.5%) 1 (4.6%)	0 (0%) 9 (90.0%) 1 (10.0%)

Abbreviations: HIV, human immunodeficiency virus; IeDEA, International epidemiology Databases to Evaluate AIDS; TB, tuberculosis.

^1^Screening for TB infection and TB disease includes use of symptom screening alone or in combination with other tools according to site protocols.

^2^Out of 131 clinics with co-located pharmacies.

^3^Out of clinics whose pharmacies had TB medications available.

### Approaches to TB Diagnosis and Associated Factors

TB Disease Screening Practices. Most ART programs (89.6%) routinely screened PLHIV for TB disease at the time of enrollment in HIV care (Table 1). Screening involved the use of TB symptom screening, according to site protocols. Almost all sites also offered regular screening for TB during follow-up HIV care visits, with most providing screening in the HIV clinic (78.5%) and others providing screening within the same facility (14.8%).

Most sites (93.3%) applied an algorithm to support TB screening for adults, children, or both. Sites used a child-specific screening algorithm for patients up to a median age of 14 years (*n* = 105, interquartile range [IQR], 12-15 years), and an adult-specific screening algorithm for patients beginning at a median minimum age of 15 years (*n* = 99, IQR, 14-16 years). TB symptoms commonly used in screening algorithms for children broadly aligned with WHO recommendations for TB screening in CLHIV, with 86.5% of sites (109/126) screening for the combination of cough, fever, poor weight gain or failure to thrive, and history of contact with a person with TB.^12^ Other sites reported screening CLHIV for some but not all of these components; poor weight gain or failure to thrive was applied most frequently (118/126, 93.7%), followed by cough (117/126, 92.9%), history of contact with a person with TB (116/126, 92.1%), and fever (113/126, 89.7%).

Overall, children had similar access to TB disease screening when compared to PLHIV of all ages. For children, screening for TB disease was provided at 88.2% of sites, applying an algorithm and/or additional diagnostic tools according to site protocols. However, availability for children varied significantly by region (range 66.7%-96.8% of sites, *P* = .004).

Use and Availability of TB Diagnostics. Expectorated sputum was the most common sample used for TB diagnosis in children at sites overall (88, 65.2%) (Table 2). Other diagnostic samples used for children included induced sputum (67, 49.5%), gastric aspirates (45, 53.3%), urine (45, 33.3%), and biopsy (52, 38.5%). The use of gastric aspirate and biopsy samples for children varied significantly by region, site setting, and level of care, while variations in the use of the other samples by site context did not approach statistical significance. Use of the string test for duodenal sampling was infrequent across all sites (10, 7.4%), with no significant variation by site context.

**Table 2.** Microbiologic Samples Used for Children at 135 Clinics Serving Children and Adolescents With HIV in the Global IeDEA Consortium Which Diagnosed TB in 2019

Program characteristic	*n*	Microbiologic sample, *n* (%)					
		Expectorated sputum	Induced sputum	Gastric aspirate	Urine	Biopsy	String test
Total	135	88 (65.2%)	67 (49.5%)	72 (53.3%)	45 (33.3%)	52 (38.5%)	10 (7.4%)
IeDEA Region Asia-Pacific Caribbean, Central and South America Central Africa East Africa Southern Africa West Africa *P*-value	17 6 17 63 22 10	11 (64.7%) 5 (83.3%) 9 (52.9%) 40 (63.5%) 16 (72.7%) 7 (70.0%) *P* = .780	12 (70.6%) 3 (50.0%) 6 (35.3%) 27 (42.9%) 14 (63.6%) 5 (50.0%) *P* = .199	13 (76.5%) 4 (66.7%) 12 (70.6%) 27 (42.9%) 7 (31.8%) 9 (90.0%) *P* = .002	5 (29.4%) 2 (33.3%) 1 (5.9%) 25 (39.7%) 10 (45.5%) 2 (20.0%) *P* = .069	13 (76.5%) 3 (50.0%) 5 (29.4%) 17 (27.0%) 9 (40.9%) 5 (50.0%) *P* = .007	1 (5.9%) 1 (16.7%) 1 (5.9%) 7 (11.1%) 0 (0%) 0 (0%) *P* = .442
Setting Urban Periurban Rural *P*-value	40 60 35	29 (72.5%) 37 (61.7%) 22 (62.9%) *P* = .508	23 (57.5%) 31 (51.7%) 13 (37.1%) *P* = .195	23 (57.5%) 38 (63.3%) 11 (31.4%) *P* = .009	15 (37.5%) 20 (33.3%) 10 (28.6%) *P* = .715	21 (52.5%) 25 (41.7%) 6 (17.1%) *P* = .006	2 (5.0%) 7 (11.7%) 1 (2.9%) *P* = .281
Level of care^1^ Health center District hospital Tertiary hospital *P*-value	68 15 45	47 (69.1%) 8 (53.3%) 30 (66.7%) *P* = .503	28 (41.2%) 7 (46.7%) 28 (62.2%) *P* = .089	25 (36.8%) 8 (53.3%) 37 (82.2%) *P* < .001	23 (33.8%) 5 (33.3%) 15 (33.3%) *P* = .998	15 (22.1%) 7 (46.7%) 29 (64.4%) *P* < .001	6 (8.8%) 0 (0%) 3 (6.7%) *P* = .703

*P*-values from Fisher’s exact tests or chi-square tests comparing use for children to non-use for children. Fisher’s exact tests were used for variables with at least one expected cell frequency < 5, and chi-square tests were used for variables with expected cell frequencies ≥ 5. Abbreviations: HIV, human immunodeficiency virus; IeDEA, International epidemiology Databases to Evaluate AIDS; TB, tuberculosis.

^1^Out of 128 clinics with available level of care designation. Tertiary hospital indicates a regional, provincial, or university hospital.

Nucleic acid amplification testing (NAAT) was used for TB diagnosis in children at 110 sites (81.5%) and available on-site at 72 sites (53.3%) (Supplementary Table S1). Sputum microscopy was used for TB diagnosis in children at 99 sites (73.3%). Mycobacterial culture was used at 70 sites (51.9%) and available on-site at 43 sites (31.9%). In total, 119 sites (88.1%) used any sputum-based diagnostics—NAAT, microscopy, and/or culture—to diagnose TB in children. Drug resistance testing was used at 63 sites (46.7%; available on-site at 29.6%). Use was more limited for urine lipoarabinomannan (LAM) (40, 29.7%), tuberculin skin tests (TST) (42, 31.1%), and interferon-gamma release assays (IGRA) (10, 7.4%), and on-site availability of these tests was also limited (37.0%, 28.1%, and 10.4%, respectively). Although most sites used chest X-rays (105, 77.8%), X-rays were available on-site at only 53.3% of sites. Whether sites charged fees for tests varied both by diagnostic tool and by region. Overall, fees were most often charged for chest X-rays (19.3%, range 0%-60.0%) compared with other tools.

Use varied diagnostic tools in different site contexts (Table 3). NAAT use was not significantly different across sites in different regions, settings, or levels of care. Sputum microscopy was used at all sites in the West Africa and Caribbean, Central and South America regions, while use was significantly less likely at sites in other regions (*P* = .022). Mycobacterial culture use was significantly more likely in urban and periurban sites (*P* = .019) and in tertiary care sites (*P* = .001) but did not vary significantly by region. Use of chest X-ray, drug resistance testing, and TST was all significantly more likely at tertiary hospital sites compared with health center and district hospital sites (*P* < .001–*P* = .003). Use of urine LAM was significantly more likely at sites in East Africa and Southern Africa compared to other regions (*P* = .007); there was no significant difference in use when examined by site setting or level of care.

**Table 3.** Diagnostic Tools Used for Children at 135 Clinics Serving Children with HIV in the Global IeDEA Consortium Which Diagnosed TB in 2019.

Program characteristic	*n*	Diagnostic tool, *n* (%)							
		AFB smear	TB NAAT	Myco-bacterial culture	Urine LAM	Drug resistance testing	TST	IGRA	Chest X-ray
Total	135	99 (73.3%)	110 (81.5%)	70 (51.9%)	40 (29.6%)	63 (46.7%)	42 (31.1%)	10 (7.4%)	105 (77.8%)
IeDEA Region Asia-Pacific Caribbean, Central and South America Central Africa East Africa Southern Africa West Africa *P*-value	17 6 17 63 22 10	13 (76.5%) 6 (100%) 8 (47.1%) 48 (76.2%) 14 (63.6%) 10 (100%) *P* = .022	14 (82.4%) 4 (66.7%) 13 (76.5%) 55 (87.3%) 14 (63.6%) 10 (100%) *P* = .076	12 (70.6%) 5 (83.3%) 8 (47.1%) 27 (42.9%) 13 (59.1%) 5 (50.0%) *P* = .199	1 (5.9%) 0 (0%) 3 (17.6%) 26 (41.3%) 9 (40.9%) 1 (10.0%) *P* = .007	14 (82.4%) 3 (50.0%) 6 (35.3%) 24 (38.1%) 13 (59.1%) 3 (30.0%) *P* = .013	12 (70.6%) 3 (50.0%) 9 (52.9%) 5 (7.9%) 6 (27.3%) 7 (70.0%) *P* < .001	6 (35.3%) 0 (0%) 1 (5.9%) 1 (1.6%) 2 (9.1%) 0 (0%) *P* = .002	14 (82.4%) 5 (83.3%) 13 (76.5%) 46 (73.0%) 17 (77.3%) 10 (100%) *P* = .566
Setting Urban Periurban Rural *P*-value	40 60 35	27 (67.5%) 43 (71.7%) 29 (82.9%) *P* = .300	31 (77.5%) 51 (85.0%) 28 (80.0%) *P* = .618	24 (60.0%) 35 (58.3%) 11 (31.4%) *P* = .019	11 (27.5%) 20 (33.3%) 9 (25.7%) *P* = .691	23 (57.5%) 29 (48.3%) 11 (31.4%) *P* = .074	20 (50.0%) 20 (33.3%) 2 (5.7%) *P* < .001	7 (17.5%) 3 (5.0%) 0 (0%) *P* = .010	33 (82.5%) 50 (83.3%) 22 (62.9%) *P* = .047
Level of care^1^ Health center District hospital Tertiary hospital *P*-value	68 15 45	51 (75.0%) 10 (66.7%) 36 (80.0%) *P* = .566	53 (77.9%) 14 (93.3%) 40 (88.9%) *P* = .246	28 (41.2%) 5 (33.3%) 33 (73.3%) *P* = .001	21 (30.9%) 6 (40.0%) 11 (24.4%) *P* = .496	26 (38.2%) 4 (26.7%) 30 (66.7%) *P* = .003	12 (17.6%) 4 (26.7%) 25 (55.6%) *P* < .001	3 (4.4%) 0 (0%) 7 (15.6%) *P* = .084	44 (64.7%) 15 (100%) 42 (93.3%) *P* < .001

*P*-values from Fisher’s exact tests or chi-square tests comparing use for children to non-use for children. Fisher’s exact tests were used for variables with at least one expected cell frequency < 5, and chi-square tests were used for variables with expected cell frequencies ≥ 5. Abbreviations: AFB, acid fast bacillus; HIV, human immunodeficiency virus; IeDEA, International epidemiology Databases to Evaluate AIDS; IGRA, interferon gamma release assay; LAM, lipoarabinomannan; NAAT, nucleic acid amplification testing; TB, tuberculosis; TST, tuberculin skin test.

^1^Out of 128 clinics with available level of care designation. Tertiary hospital indicates a regional, provincial, or university hospital.

For tests of TB infection, the overall availability of tuberculin skin testing (TST) or interferon-gamma release assay (IGRA) was reported by 31.1% and 7.4% sites, respectively. The use of these diagnostics of TB infection varied by IeDEA region. Use of TST and IGRA were more likely in urban and periurban sites compared to rural sites (*P* < .001 and *P* = .010, respectively).

### Approaches to TB Management and Prevention

TB Disease Management and Care Integration Practices. Overall, 126 sites (93.3%) provided integrated TB-HIV care with on-site TB disease treatment (Supplementary Table S2). Most of these sites (124, 98.4%) performed contact tracing for household contacts of PLHIV who were diagnosed with TB disease. Responsibility for contact tracing (by clinic staff or by a separate team) varied by region.

Sites reported a variety of follow-up approaches to reach patients with TB disease who missed appointments. Most sites (100, 79.4%) attempted to reach patients or their families by phone. Sites also used home visits, either by clinic staff (66, 52.4%) or a community outreach worker (76, 60.3%), and outreach by a peer supporter or mentor (48, 38.1%). A minority of sites (23, 18.3%) used written messaging via letter or electronic communication such as email, short message service (SMS), or online patient portal.

Use of TB Preventive Therapy TB preventive therapy (TPT) was provided for PLHIV who screened negative for TB at 108 sites (80.0%) (Supplementary Table S3). TPT provision varied significantly across regions (range 41.2%-100%, *P* < .001). Sites reported using multiple criteria to determine eligibility for TPT. Most sites provided TPT to CLHIV who were < 5 years of age (81.5% overall, range 59.1%-100%, *P* = .027). Fewer sites provided TPT to CLHIV who were 6-15 years of age (63.9% overall, *P* < .001). Other eligibility criteria included history of any TB contact (80.6%, *P* = .069), receipt of ART (75.9%, *P* < .001), new HIV diagnosis (74.1%, *P* < .001), lack of previous TPT (72.2%, *P* < .001), and previous treatment for TB disease (61.1%, *P* < .001). Only 52.8% of sites provided TPT to children with household TB contact, and differences did not vary significantly by region (range 40.0-100%, *P* = .152). A positive test of TB infection was used infrequently to guide TPT provision for children (25.9% overall, range 0-72.7%, *P* < .001), correlating with the limited use of TST and IGRA in most regions.

Six months of isoniazid (6H) was the most widely used TPT regimen in all regions (88.0% overall, range 60.0%-100%, *P* < .001). Other TPT regimens were provided to children at very few sites, including nine months of isoniazid (9H, 3.7%), 12 months of isoniazid (12H, 3.7%), 36 months or lifetime isoniazid (36H, 0.9%), 3 months of rifampin (3R, 0.9%), 4 months of rifampin (4R, 1.9%), 3 months of isoniazid and rifampin (3HR, 1.9%), 4 months of isoniazid and rifampin (4HR, 0.9%), and 12 weeks of once-weekly isoniazid and rifapentine (3HP, 0.9%). No sites provided a once-daily regimen of isoniazid and rifapentine for 1 month (1HP). Five sites in East Africa and Southern Africa (4.6% overall) reported providing TPT regimens for multidrug-resistant TB exposure.

Availability of Medications for TB Treatment or Prevention. Most sites (90.4%) provided on-site TB disease treatment for CLHIV (Table 1). Almost all sites (97.0%) had a co-located pharmacy stocking medications for TB treatment and/or prevention. At these sites, most pharmacies in all regions had available supplies of isoniazid (88.6% overall, range 60.0%-100%) and other TB medications (87.0% overall, range 60.0%-93.8%). Supplies of rifapentine were limited at co-located pharmacies in all regions (21.4% overall, range 10.0%-29.4%). Pharmacies reported medication stockouts lasting one week or longer for isoniazid (23/116, 19.8%), rifapentine (3/28, 10.7%), and other TB medications (11/114, 9.6%). Most sites (94.1%) charged no fees other than insurance copays for TB medications.

## Discussion

This study characterizes TB services for CLHIV as reported by 135 clinical facilities in 32 countries and six IeDEA regions. Across these sites in the global IeDEA consortium, integration of TB/HIV treatment for CLHIV was high. Findings include overall access to NAAT for children—which increased since a previous site assessment survey.^6^ Heterogeneity was observed across sites for several TB screening, diagnostic, management, and prevention practices. The survey revealed implementation gaps across critical areas, including access to diagnostics and to TPT. Noted gaps highlight areas in which efforts to fight childhood TB may be hampered, for which continued assessment over time is needed.

Access to any sputum-based diagnostic approached 90% for CLHIV. Approximately half of all sites, however, did not use gastric aspirate sampling or induced sputum, which are essential tools for diagnosis in children.^12^ While access to NAAT was high overall, variability was noted across regions. We note that this survey did not ask about stock-outs of Xpert cartridges or other supply challenges limiting NAAT use, which may be common and exacerbated by the COVID-19 pandemic.^13,14^ Access to TB drug resistance testing—either through mycobacterial culture or molecular resistance testing—was limited, particularly in health center and district hospital sites. Lack of access to resistance testing may preclude recognition of drug-resistant TB, resulting in under-diagnosis and disproportionate delays to effective treatment.^15-18^ Access to urine LAM, which has shown promise as a noninvasive, specific screening test for CLHIV, was also limited at the time of the survey.^19^ This survey did not ask about reasons for the use or non-use of particular tools; further exploration of these factors is needed to address implementation gaps.

Integration of HIV and TB services, including co-located TB screening and treatment, is key for supporting TB/HIV management, care coordination, and optimal outcomes.^20^ At these sites, care integration was high overall, with 90% of sites providing on-site TB screening and treatment for CLHIV. However, this may not reflect adequate care integration for children with drug-resistant TB as fewer than one-third of sites had access to TB resistance testing on-site. Medication stock-outs also occurred in all regions. Such interruptions in access to medications to treat and prevent TB may impact sites’ abilities to provide consistently integrated care.

Active case finding was widely used to identify household contacts of PLHIV who were diagnosed with TB disease, though further system integrations may be needed to ensure provision and completion of TPT for eligible contacts. Household contact tracing is a key element to stop the spread of TB. This is of acute importance for young children, for CLHIV, and for those who may be HIV-exposed and uninfected, who are all at increased risk for TB.^7,21^

Reported practices for provision of TPT were variable. Notably, only 52.8% of sites reported providing TPT to children with household TB contact. TPT provision was also low for children ages 6-15 years. The WHO recommends TPT as part of the package of care for all PLHIV (regardless of age) if TB disease is ruled out.^7^ While global provision of TPT to PLHIV has exceeded global targets, uptake varies, and CLHIV experience different barriers that contribute to lower rates of TPT initiation and completion in some settings.^1,22,23^ Further work is needed to assess barriers to implementing universal TPT for CLHIV.

Access to shorter, rifamycin-based TPT regimens was uncommon in all regions. Expanded access to shorter regimens may improve feasibility, acceptability, and completion of TPT.^24-26^ Rifamycin-based TPT regimens, however, have drug-drug interactions with some antiretroviral medications that necessitate dose adjustments, which may impact uptake for CLHIV.^7^ The WHO has recommended shorter TPT regimens since 2015, and updated guidelines supporting the use of shorter regimens for all individuals, regardless of HIV status or local TB burden, were first published in 2020.^21^ Since this survey was conducted in 2020-2021 and queried pre-pandemic practices, findings may not reflect more recent practice changes in response to evolving guidelines for TPT regimens in PLHIV.^7^ Further work is needed to assess ongoing access to shorter TPT regimens among CLHIV and any implementation barriers that may remain.

This study is strengthened by the inclusion of 135 diverse sites providing HIV treatment to children across global regions. Further, the use of a systematic process for survey development and refinement, building on previous site assessments in IeDEA and following best practices for survey design, supports the accuracy of these data. Notable limitations include that reported practices may not reflect variability in actual day-to-day practices and that we were unable to further explore reasons for differing practices. Potential differences in survey interpretation could have impacted responses; efforts to guard against this included significant processes of iterating, refining, and piloting survey items. While we queried pre-pandemic practices, active pandemic-related disruptions during the survey may have impacted responses. The IeDEA sites included—some of which may provide higher levels of care than others in their respective regions—may not be fully representative of service availability within regions.^9^ The East Africa region is also represented by a greater number of sites, with comparably fewer sites in other regions.

The survey predated recent events, including the dismantling of USAID, which consequent disruptions to TB and HIV services globally. Practices including integrated TB/HIV care and community-based TB services and follow-up may be particularly impacted. There is a need for ongoing assessment of care practices—which may not be feasible without sustained or new global health funding.

In conclusion, this global survey of HIV treatment programs in the IeDEA consortium found encouraging overall availability of NAAT and integrated TB/HIV treatment for CLHIV, but access to key tools varied by context. Heterogeneous implementation gaps remain—particularly for TB drug susceptibility testing, TPT delivery, and TPT regimens—which impede TB prevention, management, and optimal outcomes for CLHIV. The recent dismantling of US global health funding and programs may only widen implementation gaps, threatening the progress that has been made in integrated TB/HIV care. Now and as ever, attention to these gaps requires ongoing assessment over time to improve the equitable availability of critical tools for TB care and prevention for CLHIV.

## Supplementary Material

piaf050_suppl_Supplementary_Tables_S1-S3

## IeDEA Asia Pacific

Chidchon Chansilpa, Trevor Dougherty, Azar Karminia, Matthew Law, Jeremy Ross, Annette Sohn.

**Australia:** Ivette Aguirre, David Baker, Mark Bloch, Safaa Cabot, Andrew Carr, Deborah Couldwell, Sian Edwards, Beng Eu, Heather Farlow, Robert Finlayson, Manoji Gunathilake, Cherie Hazlewood, Jennifer Hoy, Julian Langton-Lockton, Jacqueline Le, Elizabeth Leprince, Ariane Minc, Richard Moore, Maree O’Sullivan, Norm Roth, Dianne Rowling, Darren Russell, Nathan Ryder, Craig Saunders, Julie Silvers, David J. Smith, David Sowden, Grant Sweeney, Lynn Tan, Ricard Teague, David Templeton, Caroline Thng, Ian Woolley. **Cambodia:** Vohith Khol, Penh Sun Ly. **China:** Tsz Hei Li, Lee Man Po. **India:** Aarti Kinikar, Nagalingeswaran Kumarasamy, Sanjay Mundhe, Sanjay Pujari, Shashikala Sangle, Smita Nimkar. **Indonesia:** Madelein Jassin, Nia Kurniati, Tuti Parwati Merati, Dina Muktiarti, Rizqi Amalia, Ni Made Dewi Dian Sukmawati, Ketut Dewi Kumara Wati, Evy Yunihastuti. **Japan:** Junko Tanuma. **Republic of Korea:** Jun Yong Choi. **Malaysia:** Raja Iskandar Shah Raja Azwa, Chan Kwai Cheng, Yasmin Mohamed Gani, Thahira Jamal Mohamed, Fong Siew Moy, Revathy Nallusamy, Mohamad Zulfahami Mohd Nor, Nuraini Rudi, Wong Peng Shyan, Nik Khairulddin Nik Yusoff. **The Philippines:** Rossana Ditangco. **Taiwan:** Yu-Jiun Chan, Pei-Chieh Wu, Ping-Feng Wu. **Thailand:** Anchalee Avihingsanon, Romanee Chaiwarith, Kulkanya Chokephaibulkit, Suwimon Khusuwan, Sasisopin Kiertiburanakul, Pope Kosalaraksa, Pagakrong Lumbiganon, Pradtana Ounchanam, Thanyawee Puthanakit, Supattra Rungmaitree, Nuttarika Solai, Tavitiya Sudjaritruk. **Vietnam:** Vu Thien An, Do Duy Cuong, Chau Viet Do, Bui Vu Huy, Tuan Quy, Kinh Van Nguyen, Luan Nguyen, Van Lam Nguyen, Yen Thi Nguyen, Vuong Minh Nong, Huu Khanh Truong, Ngo Thi Thu Tuyen.

## IeDEA Caribbean, Central and South America (CCASAnet)

Catherine C. McGowan, Stephany Duda, Fernanda Maruri, C. William Wester

**Argentina:** Florencia Cahn, Pedro Cahn, Carina Cesar, Valeria Fink, Omar Sued. **Brazil:** Lara Coelho, Daisy Maria Machado, Jorge Pinto. **Chile:** Marcelo Wolff. **Haiti:** Vanessa Rouzier. **Honduras:** Denis Padgett. **Mexico:** Brenda Crabtree Ramírez. **Peru:** Eduardo Gotuzzo.

## Central Africa IeDEA

Ellen Brazier, Denis Nash.

**Burundi:** Jérémie Biziragusenyuka, Patrick Gateretse, Pelagie Nimbona, Olive Niyonkuru, Christelle Twizere.

**Cameroon:** Rogers Ajeh, Surreng Anicetus, Amadou Djenabou, Priscilla Enow, Eyongetah Mbu, Martin Manga, Mercy Ndobe, Judith Nasah, Elle Nathalie Syntyche Ekossono, Mireille Teno Bouseko. **Democratic Republic of the Congo:** Faustin Kitetele, Patricia Lelo. **Republic of Congo**: Merlin Isidore Justin Diafouka, Adolphe Mafoua, Dominique Mahambou Nsonde. **Rwanda:** Uitonze Aime Maurice Bihira, Marie Chantal Dusabe, Rosine Feza, Jean Claude Habanabashaka, Viateur Habumuremyi, Ernestine Igizeneza, Anne Marie Kamigisha, Gallican Kubwimana, Gilbert Maniriho, Gilbert Mbaraga, Benjamin Muhoza, Jeanne Mukakarangwa, Joyce Mukamana, Patricie Mukanyirigira, Yvone Claude Mukeshimana, Athanase Munyaneza, Gad Murenzi, Jacqueline Musaninyange, Jules Ndumuhire Nyiraneza, Fidele Ntarambirwa, Marie Louise Nyiraneza, Josette Tuyishime, Yvonne Tuyishimire, Alexis Ubandutira, Florance Umugiraneza, Rosine Umugwaneza, Olive Uwamahoro, Pauline Uwamahoro, Marie Victoire Uwambaje, Clarisse Uwimpuhwe, Siphora Uwiragiye.

## East Africa IeDEA

Yee Yee Kuhn, Beverly Musick, Kara Wools-Kaloustian.

**Kenya:** Felix Adera, BeatricecAdhiambo, Khaemba Aggrey, Daniel Akadikor, Felix Ambulla, DorahApiyo, Patrick Ariya, Naftal Atemba, Fridah Ayodi., Chirchir Benard, Maureen Bett, Serafine Birgen, Rael Bwalei, Nancy Chebon, Valentine Jirry Chebor, Philip Chebuiywo, Jacline Chemutai, Emily Chepkorir, Carolyne Chepseba, John Chirchir, John Chirchir, Lameck Diero, Benard Dukwa, Alice Elphas, Tom Etyang, Agnes Idiama, Ann Jebichuko, Delvine Jepchumba, Churchill Juma, Maureen Juma, Sheila Juma., Julie Kadima, Rose Karani, Christopher Keitany, Pricilla Keter, Lucy Kiavoga, Harrison Kibet, Ruth Kimutai, Mutai Kiplagat, Wilfred Kiprono, Nicholas Kogei Kipruto, Asenath Kirimi, Zeddy Koech, Carolyne Kosgei, Karen Kutto, Mildred Kweyu, Ephraim Kenneth Liech, Milka Limo, Rose Maina, Priscah Marumbu, Agnes Masese, Patricia Mochotto, Omudeck Molly, Tom Momanyi, John W. Murutu, Praxidis Mwanda, Lillian Ndakalu, Rose N. Nderitu, Sarah Obatsa, Fredrick Obiga, Moses Oboya, Joseph Odhiambo, George Olaya, Oscar Omanyala, Christine Oray, Molly Otieno, Modesta Toto Otwane, Paul Ouma, Charles Owuor, Doris Tutu Pepela, Collins Pessah, Evans Rotich, Edwin K. Rotich, Titus C. Rutto, Monica Shikuku, Rose Naliaka Sibweche, Robert Wanyonyi Simiyu, Hellen Siria, Michael Some, Winnie Cherotich Songok, Immaculate Tanui, Grace Wafula, Rebecca Wambura, Ellah Wanjala, Carolyne Wanyama, Hellen Wanyonyi, Emmanuel Woyakapel, Wandera Zelbabel, Judy, Kiprop, Beatrice, Leah, Dominic, Tallam. **United Republic of Tanzania:** Dikengela Gwimo, Ester Kinyota, Jerome Lwali, Rita Lyamuya, Richard Machemba, Julia Mathias, Lilian Mkombachepa, Athuman Mokiwa, Ombeni Mushi, Charles Ndunguru, Kapella Ngonyani, Charles Nyaga, Happiness Ruta, Mark Urassa. **Uganda:** James Akanyihayo, Arnold Arinaitwe, Jesca Batuuka, Walusimbi Birungi, John Nyanzi Bugembe, Ahmed Ddungu, Kato Francis, Bangira Imran, George William Kafuuma, John Bosco Kalulue, Grace Kanaabi, Michale Kanyesigye, Godfery Karuhanga, Charles Kasozi, Godfrey Kasule, Assumpta Katusime, Donozio Kibalama, Donozio Kibalama, Simon Peter Kimera, Namatovu Kulusumu, Yusuf Lule, Isaac Lwanga, Margaret Mluindwa, Jemba Moses, Sseremba Mubarak, Daniel Muggaga, Evelyn Mukalazi, Joseph Muleebwa, Derick Mulema, Ivan Musisi, John Muwawu, Winnie Muyindike, Dick Mwaka, Milly Naava, Immaculate Nabiyki, Agnes Nabusulwa, Dorah Nakabugo, Esther Nakamya, Daisy Nakanwagi, Oliver Nakato, Lydian Nakayi, Patience Nakigozi, Juliet Nakku, Juliet Nakuya, Justine Nakyomu, Joan Namayanja, Sarah Namirembe, Juliet Namugumya, Ezereth Namukasa, Viola Namulindwa, Irene Nankya, Grace Mugagga Nannyondo, Harriet Nansamba, Denis Nansera, Brenda Nanyanzi, Esther Celina Nanyonjo, Irene Nayiga, Isaac Opira, Noela C. Owarwo, Sserunkuma Resty, Haruna Semuwemba, Julius Senoga, Gerald Sseguya, John Paul Ssekyewa, Matthew Ssemakadde, Jonah Tebajjwa, Doreen Tugumisirize, Robinah Tushemerirwe, Kawuki Waliyi, Fenehance, Medard.

## IeDEA North American AIDS Cohort Collaboration on Research and Design (NA-ACCORD)

Richard Moore, Keri Althoff, Aimee Freeman.

**Canada:** Jennifer Bishop, M J Gill, Mona Loutfy, Graham Smith. **United States of America:** Laura Bamford, Anthony Black, Asia Brice, Sheldon Brown, Jonathan Colasanti, Piper Duarte, Cynthia Firnhaber, Matthew Goetz, Chris Grasso, Barbara Gripshover, Michael Horberg, Rita Kelly, Ken Levine, Mitchell Luu, Vincent Marconi, Karen Maroney, Kenneth Mayer, Angel Mayor, Catherine McGowan, Richard Moore, Ami Multani, Sonia Napravnik, Ank Nijhawan, Richard Novak, Frank Palella, Maria C. Rodriguez, Mia Scott, Ellen Tedaldi, James Willig.

## IeDEA Southern Africa

Morna Cornell, Mary-Ann Davies, Matthias Egger, Andreas Haas.

**Lesotho:** Monkoe Bereng, Maleshoane Kalake, Keketso Lenela, Relebohile Seretse. **Malawi:** Matthews Chintenga, Jane Chiwoko, Joe Gumulira, Jacqueline Huwa, Rafique Maluwa, Beatrice Matanje, Ronald Mbewe, Sunshine Mfungwe, Zakaliah Mphande, Hannock Tweya. **Mozambique:** Idiovino Rafael. **South Africa:** Patti Apolles, Eunice Beneke, Siphephelo Dlamini, Claire Edson, Brian Eley, Jonathan Euvrard, Geoffrey Fatti, Bridgette Goeieman, Ashraf Grimwood, David Huang, Susan Hugo, Zahiera Ismail, Lauren Jennings, Thulile Mathenjwa, Lizette Monteith, Zamuxolo Mshweshwe, Mfundi Ntuli, EN Ndlovu, Hloniphile Ndlozi, Sylvia Noyakaza, Hans Prozesky, Helena Rabie, Nosisa Sipambo, Karl-Günter Technau, Thokozani Tembe, Nontando Xaba**. Zambia:** Thandiwe Njobvu, Mary Munthaly, Elly Mwetwa, Gillian Kabeba, Derrick Mwendafilumba, Ethel Maanguka, Nelly Manyika, Chalwe Mwansa, Future Banda, Dickson Mwenda, Abel Bwalya, Leah Shapi, Kasapo Syame, Rita Sashi, Chisha Mulenga, Ruth Nanyangwe. **Zimbabwe:** Cleophas Chimbetete, A. Chinofunga, J. Mhike, E. Mubvigwi, F. Nyika, Kumbirai Pise Quarter.

## IeDEA West Africa

Shino Chassagne Arikawa, Renaud Becquet, Charlotte Bernard, François Dabis, Sophie Desmonde, Désiré Dahourou, Didier Koumavi Ekouevi, Antoine Jaquet, Julie Jesson, Valeriane Leroy, Karen Malateste, Elodie Rabourdin, Thierry Tiendrebeogo.

**Benin:** Michée Assogba, Marcelline d’Almeida, Djimon Marcel Zannou, Ghislaine Hounhoui. **Burkina Faso:** Denise Bere, Armel Poda, Gbolo Pooda, Richard Traore. **Côte d’Ivoire:** Yao Abauble, Ouattara Abby, Patrick Acquah, Valérie Andoble, Yobo N’Dzama Aude, Jean-Claude Azani, Oka Berete, Jacques Daple Beugre, Caroline Yao Bohoussou, Simon Boni Emmanuel Brou, Henri Chenal, Abdoulaye Cissé, Nambate Coulibaly, Marie Evelyne Dainguy, Marcelle Daligou, Toni Thomas d’Aquin, Claude Desire Dasse, Madeleine Amorissani Folquet, Guy Gnepa, Olivier Gobe, Salif Guira, Denise Hawerlander, Apollinaire Horo, Guillaume Kanga, Zobo Konan Eugène Messou, Kla Albert Minga, Raoul Moh, Marie Sylvie N’Gbeche, Patricia Ogbo, Mathieu Oulai, SE Stéphanie, Tanoh Eboua, Itchy Max Valère. **Ghana:** Adwoa Kumiwa Asare Afrane, Esther Akrofi, John Christian Andoh, Lorna Renner. **Mali:** Awa Bagayoko, Kadidiatou Bagayoko, Abdou Salam Bah, Alima Berthe, Boureïma Coulibaly, Fatimata Coulibaly, Yacouba Aba Coulibaly, Aïssata Diakité, Fatoumata Bocoum, Fatoumata Boré, Fatoumata Dicko, Odile Koné, Mariam Sylla, Assitan Tangara, Mamadou Traoré. **Senegal:** Moussa Seydi. **Togo:** Edmond Amegatse, Julienne Djossou, Elom Takassi, Sénam Palanga.

**Funding:** The International Epidemiology Databases to Evaluate AIDS (IeDEA) is supported by the U.S. National Institutes of Health’s National Institute of Allergy and Infectious Diseases, the *Eunice Kennedy Shriver* National Institute of Child Health and Human Development, the National Cancer Institute, the National Institute of Mental Health, the National Institute on Drug Abuse, the National Heart, Lung, and Blood Institute, the National Institute on Alcohol Abuse and Alcoholism, the National Institute of Diabetes and Digestive and Kidney Diseases, and the Fogarty International Center: Asia-Pacific, U01AI069907; CCASAnet, U01AI069923; Central Africa, U01AI096299; East Africa, U01AI069911; NA-ACCORD, U01AI069918; Southern Africa, U01AI069924; West Africa, U01AI069919. Informatics resources are supported by the Harmonist project, R24AI24872. This work is solely the responsibility of the authors and does not necessarily represent the official views of any of the institutions mentioned above.
