# Supplementary material for: Tuberculosis Diagnosis, Treatment, and Prevention Services for Children Living with HIV in Low- and Middle-Income Countries: A Multiregional Site Survey
Source: J Pediatric Infect Dis Soc. 2025 May 28;14(6):piaf050. doi: 10.1093/jpids/piaf050 (PMC12257623; doi:10.1093/jpids/piaf050)
Supplement: piaf050_suppl_Supplementary_Tables_S1-S3 [file piaf050_suppl_supplementary_tables_s1-s3.docx]

**Tuberculosis diagnosis, treatment, and prevention services for children living with HIV in low- and middle-income countries: a multiregional site survey**

**Supplementary Tables 1 – 3**

**Supplementary Table 1.** Use and availability of diagnostic tools at 135 clinics serving children living with HIV in the global IeDEA Consortium which diagnosed TB in 2019 – Overall and by IeDEA Regions.

| **Diagnostic Tool *n* (%)** | **All**  **(*n* = 135)** | **Asia-Pacific**  **(*n* = 17)** | **Caribbean, Central and South America**  **(*n* = 6)** | **Central Africa**  **(*n* = 17)** | **East Africa**  **(*n* = 63)** | **Southern Africa**  **(*n* = 22)** | **West Africa**  **(*n* = 10)** |
| --- | --- | --- | --- | --- | --- | --- | --- |
| AFB smear  Used in TB diagnosis  Children only  Children and adults  Adults only  None  Available on-site  Fee charged for test | 10 (7.4%)  89 (65.9%)  18 (13.3%)  18 (13.3%)  101 (74.8%)  5 (3.7%) | 5 (29.4%)  8 (47.1%)  3 (17.7%)  1 (5.9%)  16 (94.1%)  1 (5.9%) | 0 (0%)  6 (100%)  0 (0%)  0 (0%)  6 (100%)  0 (0%) | 0 (0%)  8 (47.1%)  3 (17.7%)  6 (35.3%)  6 (35.3%)  2 (11.8%) | 0 (0%)  48 (76.2%)  9 (14.3%)  6 (9.5%)  54 (85.7%)  1 (1.6%) | 1 (4.6%)  13 (59.1%)  3 (13.6%)  5 (22.7%)  14 (63.6%)  0 (0%) | 4 (40.0%)  6 (60.0%)  0 (0%)  0 (0%)  5 (50.0%)  1 (10.0%) |
| TB NAAT  Used in TB diagnosis  Children only  Children and adults  Adults only  None  Available on-site  Fee charged for test | 10 (7.4%)  100 (74.1%)  20 (14.8%)  5 (3.7%)  72 (53.3%)  5 (3.7%) | 5 (29.4%)  9 (52.9%)  3 (17.7%)  0 (0%)  16 (94.1%)  2 (11.8%) | 0 (0%)  4 (66.7%)  0 (0%)  2 (33.3%)  2 (33.3%)  0 (0%) | 0 (0%)  13 (76.5%)  4 (23.5%)  0 (0%)  7 (41.2%)  1 (5.9%) | 0 (0%)  55 (87.3%)  8 (12.7%)  0 (0%)  25 (39.7%)  1 (1.6%) | 1 (4.6%)  13 (59.1%)  5 (22.7%)  3 (13.6%)  16 (72.7%)  0 (0%) | 4 (40.0%)  6 (60.0%)  0 (0%)  0 (0%)  6 (60.0%)  1 (10.0%) |
| Mycobacterial culture  Used in TB diagnosis  Children only  Children and adults  Adults only  None  Available on-site  Fee charged for test | 7 (5.2%)  63 (46.7%)  16 (11.9%)  49 (36.3%)  43 (31.9%)  9 (6.7%) | 3 (17.7%)  9 (52.9%)  3 (17.7%)  2 (11.8%)  13 (76.5%)  3 (17.7%) | 0 (0%)  5 (83.3%)  0 (0%)  1 (16.7%)  4 (66.7%)  0 (0%) | 0 (0%)  8 (47.1%)  2 (11.8%)  7 (41.2%)  3 (17.7%)  2 (11.8%) | 0 (0%)  27 (42.9%)  4 (6.4%)  32 (50.8%)  7 (11.1%)  1 (1.6%) | 1 (4.6%)  12 (54.6%)  5 (22.7%)  4 (18.2%)  12 (54.5%)  0 (0%) | 3 (30.0%)  2 (20.0%)  2 (20.0%)  3 (30.0%)  4 (40.0%)  3 (30.0%) |
| Urine lipoarabinomannan  Used in TB diagnosis  Children only  Children and adults  Adults only  None  Available on-site  Fee charged for test | 2 (1.5%)  38 (28.2%)  13 (9.6%)  82 (60.7%)  50 (37.0%)  2 (1.5%) | 0 (0%)  1 (5.9%)  0 (0%)  16 (94.1%)  1 (5.9%)  1 (5.9%) | 0 (0%)  0 (0%)  0 (0%)  6 (100%)  0 (0%)  0 (0%) | 0 (0%)  3 (17.7%)  0 (0%)  14 (82.4%)  3 (17.6%)  0 (0%) | 1 (1.6%)  25 (39.7%)  11 (17.5%)  26 (41.3%)  35 (55.6%)  0 (0%) | 0 (0%)  9 (40.9%)  2 (9.1%)  11 (50.0%)  10 (45.5%)  0 (0%) | 1 (10.0%)  0 (0%)  0 (0%)  9 (90.0%)  1 (10.0%)  1 (10.0%) |
| Drug resistance testing  Used in TB diagnosis  Children only  Children and adults  Adults only  None  Available on-site  Fee charged for test | 9 (6.7%)  54 (40.0%)  18 (13.3%)  54 (40.0%)  40 (29.6%)  6 (4.4%) | 6 (35.3%)  8 (47.1%)  3 (17.7%)  0 (0%)  11 (64.7%)  3 (17.7%) | 0 (0%)  3 (50.0%)  1 (16.7%)  2 (33.3%)  3 (50.0%)  0 (0%) | 0 (0%)  6 (35.3%)  1 (5.9%)  10 (58.8%)  4 (23.5%)  2 (11.8%) | 0 (0%)  24 (38.1%)  9 (14.3%)  30 (47.6%)  8 (12.7%)  0 (0%) | 1 (4.6%)  12 (54.5%)  4 (18.2%)  5 (22.7%)  12 (54.5%)  0 (0%) | 2 (20.0%)  1 (10.0%)  0 (0%)  7 (70.0%)  2 (20.0%)  1 (10.0%) |
| Tuberculin skin test  Used in TB diagnosis  Children only  Children and adults  Adults only  None  Available on-site  Fee charged for test | 23 (17.0%)  19 (14.1%)  5 (3.7%)  88 (65.2%)  38 (28.1%)  10 (7.4%) | 6 (35.3%)  6 (35.3%)  1 (5.9%)  4 (23.5%)  13 (76.5%)  1 (5.9%) | 0 (0%)  3 (50.0%)  0 (0%)  3 (50.0%)  3 (50.0%)  0 (0%) | 4 (23.5%)  5 (29.4%)  0 (0%)  8 (47.1%)  4 (23.5%)  5 (29.4%) | 3 (4.8%)  2 (3.2%)  2 (3.2%)  56 (88.9%)  4 (6.4%)  1 (1.6%) | 3 (13.6%)  3 (13.6%)  2 (9.1%)  14 (63.6%)  11 (50.0%)  0 (0%) | 7 (70.0%)  0 (0%)  0 (0%)  3 (30.0%)  3 (30.0%)  3 (30.0%) |
| IGRA  Used in TB diagnosis  Children only  Children and adults  Adults only  None  Available on-site  Fee charged for test | 4 (3.0%)  6 (4.4%)  8 (5.9%)  117 (86.7%)  14 (10.4%)  10 (7.4%) | 2 (11.8%)  4 (23.5%)  4 (23.5%)  7 (41.2%)  9 (52.9%)  8 (47.1%) | 0 (0%)  0 (0%)  0 (0%)  6 (100%)  0 (0%)  0 (0%) | 1 (5.9%)  0 (0%)  0 (0%)  16 (94.1%)  0 (0%)  1 (5.9%) | 0 (0%)  1 (1.6%)  2 (3.2%)  60 (95.2%)  2 (3.2%)  0 (0%) | 1 (4.6%)  1 (4.6%)  2 (9.1%)  18 (81.8%)  3 (13.6%)  0 (0%) | 0 (0%)  0 (0%)  0 (0%)  10 (100%)  0 (0%)  1 (10.0%) |
| Chest X-ray  Used in TB diagnosis  Children only  Children and adults  Adults only  None  Available on-site  Fee charged for test | 12 (8.9%)  93 (68.9%)  15 (11.1%)  15 (11.1%)  72 (53.3%)  26 (19.3%) | 5 (29.4%)  9 (52.9%)  3 (17.7%)  0 (0%)  17 (100%)  2 (11.8%) | 0 (0%)  5 (83.3%)  0 (0%)  1 (16.7%)  5 (83.3%)  0 (0%) | 0 (0%)  13 (76.5%)  2 (11.8%)  2 (11.8%)  6 (35.3%)  6 (35.3%) | 2 (3.2%)  44 (69.8%)  8 (12.7%)  9 (14.3%)  24 (38.0%)  10 (15.9%) | 1 (4.6%)  16 (72.7%)  2 (9.1%)  3 (13.6%)  12 (54.5%)  2 (9.1%) | 4 (40.0%)  6 (60.0%)  0 (0%)  0 (0%)  8 (80.0%)  6 (60.0%) |

Abbreviations: AFB, acid fast bacillus; HIV, human immunodeficiency virus; IeDEA, International epidemiology Databases to Evaluate AIDS; IGRA, interferon gamma release assay; NAAT, nucleic acid amplification testing; TB, tuberculosis.

**Supplementary Table 2.** TB management and support services available at 126 clinics with on-site TB disease treatment and serving children with HIV in the global IeDEA Consortium which diagnosed TB in 2019 – Overall and by IeDEA Regions.

| **Characteristic *n* (%)** | **All**  **(*n* = 126)** | **Asia-Pacific**  **(*n* = 16)** | **Caribbean, Central and South America**  **(*n* = 5)** | **Central Africa**  **(*n* = 15)** | **East Africa**  **(*n* = 62)** | **Southern Africa**  **(*n* = 20)** | **West Africa**  **(*n* = 8)** |
| --- | --- | --- | --- | --- | --- | --- | --- |
| Contact tracing for household contacts of HIV patients diagnosed with TB disease  Contact tracing performed  Recorded by site staff in a  dedicated TB contact register  Recorded by site staff but not in  a dedicated register  Performed by a separate public  health team  No tracing or systematic  documentation of contacts  Confirmation of TB disease  screening and TPT treatment  provision for contacts  Recorded by site staff in a  dedicated TB contact register  Recorded by site staff but not in  a dedicated register  Performed by a separate public  health team  No systematic documentation  for contacts  Confirmation of complete TPT  treatment for contacts  Recorded by site staff in a  dedicated TB contact register  Recorded by site staff but not in  a dedicated register  Performed by a separate public  health team  No systematic documentation  for contacts | 70 (55.6%)  15 (11.9%)  39 (31.0%)  2 (1.6%)  85 (67.5%)  12 (9.5%)  16 (12.7%)  13 (10.3%)  85 (67.5%)  11 (8.7%)  19 (15.1%)  11 (8.7%) | 1 (6.3%)  3 (18.8%)  12 (75.0%)  0 (0%)  5 (31.3%)  6 (37.5%)  5 (31.3%)  0 (0%)  6 (37.5%)  4 (25.0%)  6 (37.5%)  0 (0%) | 2 (40.0%)  1 (20.0%)  2 (40.0%)  0 (0%)  1 (20.0%)  0 (0%)  4 (80.0%)  0 (0%)  1 (20.0%)  0 (0%)  4 (80.0%)  0 (0%) | 10 (66.7%)  1 (6.7%)  3 (20.0%)  1 (6.7%)  10 (66.7%)  0 (0%)  2 (13.3%)  3 (20.0%)  10 (66.7%)  0 (0%)  2 (13.3%)  3 (20.0%) | 42 (67.7%)  5 (8.1%)  15 (24.2%)  0 (0%)  53 (85.5%)  4 (6.5%)  1 (1.6%)  4 (6.5%)  55 (88.7%)  4 (6.5%)  1 (1.6%)  2 (3.2%) | 12 (60.0%)  1 (5.0%)  7 (35.0%)  0 (0%)  11 (55.0%)  2 (10.0%)  2 (10.0%)  5 (25.0%)  9 (45.0%)  3 (15.0%)  4 (20.0%)  4 (20.0%) | 3 (37.5%)  4 (50.0%)  0 (0%)  1 (12.5%)  5 (62.5%)  0 (0%)  2 (25.0%)  1 (12.5%)  4 (50.0%)  0 (0%)  2 (25.0%)  2 (25.0%) |
| Follow-up approaches for patients with TB disease who miss appointments^1^  No follow-up  Phone call to individual and/or family  Message via letter, email, SMS, or  online patient portal  Home visit by clinic staff  Home visit by community outreach  worker  Outreach by peer supporter or  mentor  Other | 2 (1.6%)  100 (79.4%)  23 (18.3%)  66 (52.4%)  76 (60.3%)  48 (38.1%)  3 (2.4%) | 0 (0%)  13 (81.3%)  4 (25.0%)  3 (18.8%)  5 (31.3%)  2 (12.5%)  1 (6.3%) | 0 (0%)  5 (100%)  2 (40.0%)  3 (60.0%)  2 (40.0%)  0 (0%)  0 (0%) | 0 (0%)  12 (80.0%)  3 (20.0%)  11 (73.3%)  10 (66.7%)  2 (13.3%)  0 (0%) | 0 (0%)  45 (72.6%)  12 (19.4%)  42 (67.7%)  42 (67.7%)  35 (56.5%)  1 (1.6%) | 1 (5.0%)  18 (90.0%)  2 (10.0%)  7 (35.0%)  17 (85.0%)  9 (45.0%)  1 (5.0%) | 1 (12.5%)  7 (87.5%)  0 (0%)  0 (0%)  0 (0%)  0 (0%)  0 (0%) |
| Definition of loss to follow-up  Treatment interruption for >2 weeks  Treatment interruption for >1 month  Treatment interruption for >2 months  Treatment interruption for >3 months  Other  Not known | 38 (30.2%)  25 (19.8%)  44 (34.9%)  9 (7.1%)  2 (1.6%)  8 (6.4%) | 4 (25.0%)  3 (18.8%)  2 (12.5%)  1 (6.3%)  1 (6.3%)  5 (31.3%) | 1 (20.0%)  2 (40.0%)  2 (40.0%)  0 (0%)  0 (0%)  0 (0%) | 7 (46.7%)  0 (0%)  7 (46.7%)  0 (0%)  0 (0%)  1 (6.7%) | 19 (30.7%)  17 (27.4%)  20 (32.3%)  6 (9.7%)  0 (0%)  0 (0%) | 5 (25.0%)  3 (15.0%)  8 (40.0%)  1 (5.0%)  1 (5.0%)  2 (10.0%) | 2 (25.0%)  0 (0%)  5 (62.5%)  1 (12.5%)  0 (0%)  0 (0%) |

Abbreviations: HIV, human immunodeficiency virus; IeDEA, International epidemiology Databases to Evaluate AIDS; SMS, short message service; TB, tuberculosis.

^1^More than one response was possible.

**Supplementary Table 3.** TB Preventive Therapy (TPT) Use at 135 clinics serving children with HIV in the global IeDEA Consortium which diagnosed TB in 2019 – Overall and by IeDEA Regions.

| **Characteristic *n* (%)** | **All**  **(*n* = 135)** | **Asia-Pacific**  **(*n* = 17)** | | **Caribbean, Central and South America**  **(*n* = 6)** | | **Central Africa**  **(*n* = 17)** | | **East Africa**  **(*n* = 63)** | | **Southern Africa**  **(*n* = 22)** | | **West Africa**  **(*n* = 10)** | |
| --- | --- | --- | --- | --- | --- | --- | --- | --- | --- | --- | --- | --- | --- |
| Provision of TPT for patients who screened negative for TB | 108 (80.0%) | 11 (64.7%) | | 3 (50.0%) | | 7 (41.2%) | | 60 (95.2%) | | 22 (100%) | | 5 (50.0%) | |
| Eligibility criteria used for TPT provision^1^  Newly diagnosed with HIV  Ages <5 years  Ages 6-15 years  Receiving ART  Not previously receiving TPT  Previously treated for TB disease  Positive TST or IGRA  History of any TB contact  Household contacts, regardless of TST  or IGRA status | 80 (74.1%)  88 (81.5%)  69 (63.9%)  82 (75.9%)  78 (72.2%)  66 (61.1%)  28 (25.9%)  87 (80.6%)  57 (52.8%) | 6 (54.5%)  8 (72.7%)  6 (54.5%)  6 (54.5%)  6 (54.5%)  4 (36.4%)  8 (72.7%)  8 (72.7%)  9 (81.8%) | | 1 (33.3%)  3 (100%)  1 (33.3%)  1 (33.3%)  0 (0%)  1 (33.3%)  2 (66.7%)  3 (100.0%)  3 (100.0%) | | 5 (71.4%)  6 (85.7%)  5 (71.4%)  4 (57.1%)  3 (42.9%)  4 (57.1%)  0 (0%)  5 (71.4%)  3 (42.9%) | | 53 (88.3%)  55 (91.7%)  49 (81.7%)  56 (93.3%)  55 (91.7%)  47 (78.3%)  17 (28.3%)  50 (83.3%)  28 (46.7%) | | 14 (63.6%)  13 (59.1%)  7 (31.8%)  15 (68.2%)  13 (59.1%)  10 (45.5%)  1 (4.5%)  18 (81.8%)  12 (54.5%) | | 1 (20.0%)  3 (60.0%)  1 (20.0%)  0 (0%)  1 (20.0%)  0 (0%)  0 (0%)  3 (60.0%)  2 (40.0%) | |
| Contraindication screening prior to  TPT initiation^1^  None  Jaundice or liver disease  Peripheral neuropathy (numbness,  tingling)  Previous adverse reaction  Alcohol misuse  Age  TB disease  Other | 3 (2.8%)  95 (88.0%)  73 (67.6%)  83 (76.9%)  53 (49.1%)  23 (21.3%)  98 (90.7%)  4 (3.7%) | 2 (18.2%)  7 (63.6%)  6 (54.6%)  6 (54.6%)  2 (18.2%)  1 (9.1%)  8 (72.7%)  0 (0%) | | 0 (0%)  3 (100%)  1 (33.3%)  0 (0%)  1 (33.3%)  1 (33.3%)  2 (66.7%)  0 (0%) | | 0 (0%)  6 (85.7%)  2 (28.6%)  6 (85.7%)  4 (57.1%)  0 (0%)  6 (85.7%)  0 (0%) | | 0 (0%)  58 (96.7%)  52 (86.7%)  53 (88.3%)  33 (55.0%)  20 (33.3%)  58 (96.7%)  3 (5.0%) | | 1 (4.6%)  17 (77.3%)  12 (54.6%)  17 (77.3%)  13 (59.1%)  1 (4.6%)  19 (86.4%)  1 (4.6%) | | 0 (0%)  4 (80.0%)  0 (0%)  1 (20.0%)  0 (0%)  0 (0%)  5 (100%)  0 (0%) | |
| Adverse event monitoring while  receiving TPT^1^  None  Hepatitis symptoms (nausea, vomiting,  abdominal pain)  Peripheral neuropathy (numbness,  tingling)  Elevated liver enzymes  Flu-like symptoms  Rash  Other | 4 (3.7%)  95 (88.0%)  90 (83.3%)  73 (67.6%)  35 (32.4%)  82 (75.9%)  2 (1.9%) | 0 (0%)  11 (100.0%)  10 (90.9%)  9 (81.8%)  5 (45.5%)  9 (81.8%)  0 (0%) | | 0 (0%)  3 (100%)  1 (33.3%)  1 (33.3%)  0 (0%)  2 (66.7%)  0 (0%) | | 0 (0%)  7 (100%)  4 (57.1%)  3 (42.9%)  0 (0%)  6 (85.7%)  0 (0%) | | 0 (0%)  53 (88.3%)  57 (95.0%)  46 (76.7%)  27 (45.0%)  48 (80.0%)  0 (0%) | | 4 (18.2%)  16 (72.7%)  17 (77.3%)  10 (45.5%)  2 (9.1%)  16 (72.7%)  0 (0%) | | 0 (0%)  5 (100%)  1 (20.0%)  4 (80.0%)  1 (20.0%)  1 (20.0%)  2 (40.0%) | |
| TPT regimens provided to children^1,2^  6-month isoniazid (6H)  9-month isoniazid (9H)  12-month isoniazid (12H)  36-month/lifetime isoniazid (36H)  3-month rifampicin (3R)  4-month rifampicin (4R)  3-month isoniazid-rifampicin (3HR)  4-month isoniazid-rifampicin (4HR)  Once-weekly isoniazid-rifapentine for 12  weeks (3HP)  Once-daily isoniazid-rifapentine for 1  month (1HP)  Regimens for MDR-TB exposure | 95 (88.0%)  4 (3.7%)  4 (3.7%)  1 (0.9%)  1 (0.9%)  2 (1.9%)  3 (2.8%)  1 (0.9%)  1 (0.9%)  0 (0%)  5 (4.6%) | | 9 (81.8%)  2 (18.2%)  1 (9.1%)  0 (0%)  0 (0%)  0 (0%)  1 (9.1%)  0 (0%)  0 (0%)  0 (0%)  1 (9.1%) | | 3 (100%)  1 (33.3%)  1 (33.3%)  1 (33.3%)  1 (33.3%)  1 (33.3%)  0 (0%)  0 (0%)  0 (0%)  0 (0%)  0 (0%) | | 7 (100%)  1 (14.3%)  0 (0%)  0 (0%)  0 (0%)  0 (0%)  1 (14.3%)  0 (0%)  0 (0%)  0 (0%)  0 (0%) | | 54 (90.0%)  0 (0%)  0 (0%)  0 (0%)  0 (0%)  0 (0%)  0 (0%)  1 (1.7%)  0 (0%)  0 (0%)  1 (1.7%) | | 19 (86.4%)  0 (0%)  2 (9.1%)  0 (0%)  0 (0%)  1 (4.5%)  1 (4.5%)  0 (0%)  1 (4.5%)  0 (0%)  3 (13.6%) | | 3 (60.0%)  0 (0%)  0 (0%)  0 (0%)  0 (0%)  0 (0%)  0 (0%)  0 (0%)  0 (0%)  0 (0%)  0 (0%) |
| Follow-up approaches for patients receiving TPT who miss appointments^1,3^  No follow-up  Phone call to individual and/or family  Message via letter, email, SMS, or  online patient portal  Home visit by clinic staff  Home visit by community outreach  worker  Outreach by peer supporter or mentor  Other | 7 (6.5%)  96 (88.9%)  25 (23.2%)  46 (42.6%)  56 (51.9%)  44 (40.7%)  2 (1.9%) | | 2 (18.2%)  8 (72.7 %)  3 (27.3%)  0 (0%)  2 (18.2%)  1 (9.1%)  0 (0%) | | 0 (0%)  2 (66.7%)  1 (33.3%)  0 (0%)  1 (33.3%)  0 (0%)  0 (0%) | | 1 (14.3%)  6 (85.7%)  1 (14.3%)  5 (71.4%)  2 (28.6%)  1 (14.3%)  0 (0%) | | 0 (0%)  59 (98.3%)  16 (26.7%)  34 (56.7%)  39 (65.0%)  39 (65.0%)  0 (0%) | | 4 (18.2%)  16 (72.7%)  1 (4.6%)  5 (22.7%)  11 (50.0%)  2 (9.1%)  2 (9.1%) | | 0 (0%)  5 (100%)  3 (60.0%)  2 (40.0%)  1 (20.0%)  1 (20.0%)  0 (0%) |

Abbreviations: ART, antiretroviral therapy; HIV, human immunodeficiency virus; IeDEA, International epidemiology Databases to Evaluate AIDS; IGRA, interferon gamma release assay; MDR, multidrug resistant; SMS, short message service; TB, tuberculosis; TPT, tuberculosis preventive therapy; TST, tuberculin skin test.

^1^Out of 108 clinics that provided TPT.

^2^Some clinics did not provide TPT to children.

^3^More than one response was possible.
